# High blood pressure levels and cardiovascular risk among Munduruku indigenous people^*^

**DOI:** 10.1590/1518-8345.4970.3477

**Published:** 2021-09-03

**Authors:** Neuliane Melo Sombra, Hanna Lorena Moraes Gomes, António Manuel Sousa, Gilsirene Scantelbury de Almeida, Zilmar Augusto de Souza, Noeli das Neves Toledo

**Affiliations:** 1 Universidade Federal do Amazonas, Escola de Enfermagem de Manaus, Manaus, AM, Brazil.; 2 Scholarship holder at the Coordenação de Aperfeiçoamento de Pessoal de Nível Superior (CAPES), Brazil.; 3 Scholarship holder at the Fundação de Amparo à Pesquisa do Estado do Amazonas (FAPEAM), Brazil.; 4 Universidade do Estado do Amazonas, Escola Superior de Ciências da Saúde, Manaus, AM, Brazil.

**Keywords:** Risk Factors, Prehypertension, Hypertension, Cardiovascular Diseases, Indigenous Peoples, Nursing Research, Fatores de Risco, Pré-Hipertensão, Hipertensão, Doenças Cardiovasculares, Povos Indígenas, Pesquisa em Enfermagem, Factores de Riesgo, Prehipertensión, Hipertensión; Enfermedades Cardiovasculares, Pueblos Indígenas, Investigación en Enfermería

## Abstract

**Objective:**

to identify the risk factors associated with prehypertension and arterial hypertension among Munduruku indigenous people in the Brazilian Amazon.

**Method:**

a cross-sectional study carried out with 459 Munduruku indigenous people selected by means of stratified random sampling. Sociodemographic variables, habits and lifestyles, anthropometric data, fasting glucose and lipid profiles were evaluated. An automatic device calibrated and validated to measure blood pressure was used. The analyses of the data collected were carried out in the R software, version 3.5.1. For continuous variables, the Kruskall-Wallis test was used; for the categorical ones, Fischer’s Exact. The significance level was set at 5% and p-value≤0.05.

**Results:**

the prevalence of altered blood pressure levels was 10.2% for values suggestive of hypertension and 4.1% for pre-hypertension. The risk of prehypertension among indigenous people was associated with being male (OR=1.65; 95% CI=0.65-4.21) and having a substantially increased waist circumference (OR=7.82; 95% CI=1.80-34.04). Regarding the risk for arterial hypertension, it was associated with age (OR=1.09; 95% CI=1.06-1.12), with increased waist circumference (OR=3.89; 95% CI=1.43-10, 54) and with substantially increased waist circumference (OR=5.46; 95% CI=1.78-16.75).

**Conclusion:**

among Munduruku indigenous people, men were more vulnerable to developing hypertension; age and increased waist circumference proved to be strong cardiovascular risk factors.

## Abstract (pt)

**Objetivo:**

identificar os fatores de risco associados à préhipertensão e hipertensão arterial entre indígenas Munduruku da Amazônia brasileira.

**Método:**

estudo transversal realizado com 459 indígenas Munduruku selecionados por meio de amostragem aleatória estratificada. Foram avaliadas variáveis sociodemográficas, hábitos e estilos de vida, dados antropométricos, glicemia de jejum e perfis lipídicos. Utilizou-se aparelho automático calibrado e validado para medir a pressão arterial. As análises dos dados coletados foram realizadas pelo *software* R versão 3.5.1. Para as variáveis contínuas, utilizou-se o teste Kruskall-Wallis; para as categóricas, o Exato de Fischer. Considerou-se nível de significância de 5% e valor p≤0,05.

**Resultados:**

a prevalência de níveis pressóricos alterados foi de 10,2% para valores sugestivos de hipertensão e de 4,1% para pré-hipertensão. O risco de pré-hipertensão entre indígenas associou-se a ser do sexo masculino (OR=1,65; IC95% 0,65-4,21) e ter circunferência da cintura aumentada substancialmente (OR=7,82; IC95% 1,80-34,04). Quanto ao risco para hipertensão arterial, associou-se à idade (OR=1,09; IC95% 1,06-1,12), à circunferência da cintura aumentada (OR=3,89; IC95% 1,43-10,54) e à circunferência da cintura aumentada substancialmente (OR=5,46; IC95% 1,78-16,75).

**Conclusão:**

entre indígenas Munduruku, os homens estavam mais vulneráveis para desenvolver hipertensão; a idade e a circunferência da cintura aumentada mostraram-se como fortes fatores de risco cardiovascular.

## Abstract (es)

**Objetivo:**

identificar los factores de riesgo asociados con la prehipertensión y la hipertensión arterial entre los indígenas Munduruku en la Amazonía brasileña.

**Método:**

estudio transversal realizado con 459 indígenas Munduruku seleccionados mediante muestreo aleatorio estratificado. Se evaluaron variables sociodemográficas, hábitos y estilos de vida, datos antropométricos, glucosa en ayunas y perfiles lipídicos. Se utilizó un dispositivo automático calibrado y validado para medir la presión arterial. Los análisis de los datos recopilados se llevaron a cabo mediante el *software* R versión 3.5.1. Para las variables continuas se utilizó la prueba de KruskalWallis; para las categóricas, Exacto de Fischer. El nivel de significancia se estableció en 5% y p≤0,05.

**Resultados:**

la prevalencia de niveles alterados de presión arterial fue del 10,2% para valores sugestivos de hipertensión y del 4,1% para prehipertensión. El riesgo de prehipertensión entre los indígenas se asoció al sexo masculino (OR=1,65; IC95% 0,65-4,21) y a un aumento sustancial de la circunferencia de cintura (OR=7,82; IC95% 1,80-34,04). En cuanto al riesgo de hipertensión arterial, se asoció con la edad (OR=1,09; IC95% 1,06-1,12), con un aumento de la circunferencia de la cintura (OR=3,89; IC95% 1,43-10,54) y con un aumento sustancial de la circunferencia de la cintura (OR=5,46; IC95% 1,78-16,75).

**Conclusión:**

entre los indios Munduruku, los hombres eran más vulnerables a desarrollar hipertensión, la edad y el aumento de la circunferencia de la cintura demostraron ser fuertes factores de riesgo cardiovascular.

## Introduction

Developing countries, such as Brazil, Chile and Mexico, have undergone a rapid epidemiological transition of the infectious diseases to chronic non-communicable diseases (CNCDs), due to increased exposure to risk factors. CNCDs are responsible for more than 70% of the adult deaths worldwide, with cardiovascular diseases being the main causes of death^(1)^.

The associated risk factors, such as alcohol consumption, obesity, smoking, non-practice of physical exercise and inadequate diet, contribute to the development of diseases such as systemic arterial hypertension (SAH), which is characterized by systolic pressure values ≥ 40 and diastolic pressure values ≥ 90 millimeters of mercury (mmHg)^(2)^.

SAH is one of the most prevalent modifiable cardiovascular risk factors in the world. It is characterized as a CNCD^(3)^ often associated with metabolic disorders and to functional and/or structural changes in target organs. It is aggravated by the presence of dyslipidemia, abdominal obesity, glucose intolerance and diabetes *mellitus* (DM).

Pre-hypertension, characterized by systolic values of 130-139 mmHg and/or diastolic values of 85-89 mmHg, is also worrisome within the scenario of risk factors, as it is associated with a higher risk of developing SAH and cardiac diseases. It is known that nearly one third of the cardiovascular events related to high blood pressure occur in individuals who are pre-hypertensive^(2)^.

When comparing countries from an income perspective, it is noticed that the prevalence of hypertension is higher in low-income countries (31.5%) than in high-income countries (28.5%)^(4)^. In Brazil, in 2018, among the non-indigenous population, the frequency of adults who reported a medical diagnosis of hypertension varied between 15.9% in São Luís and 31.2% in Rio de Janeiro. In the group of 27 cities in the country, the frequency of medical diagnosis of arterial hypertension was 24.7%, being higher among women (27.0%) than among men (22.1%)^(5)^.

Among the indigenous peoples, the economic, social, cultural and environmental changes that have occurred in the world have caused several harms to the individual and collective health of the different ethnic groups, highlighting the greater vulnerability for the development of chronic diseases^(6)^. A number of studies show that the cardiovascular disease (CVD) risk factors are more prevalent among some ethnic minorities, people of lower socioeconomic status and rural populations in most Latin American countries^(1,7)^.

The increase in the number of cases of CNCDs and their aggravations has been more frequent among indigenous peoples in all the countries. A descriptive cross-sectional study carried out in Mexico, with 2,596 indigenous people from different ethnic groups, revealed that the prevalence of hypertension was 42.7%^(8)^.

Among the Brazilian indigenous people, the presence of SAH has been increasingly observed, with a 6.2% increase in prevalence in the last 4 decades and metaregression, indicating that the chance of a Brazilian indigenous person developing hypertension is 12%^(9)^. The Mura indigenous people, who live in the Amazon region on the banks of the Madeira River and of the Murutinga Lake, have a 26.6% prevalence of hypertension, a similar percentage to that of non-indigenous people. This fact indicates that the cardiovascular risk factors have been growing at an accelerated pace also in this ethnic group^(10)^.

The Brazilian North region has the largest number of indigenous inhabitants. In 2010, this number corresponded to 342,836 The five municipalities that presented the largest self-declared indigenous population in Brazil belonged to the state of Amazonas. It is noteworthy that the Munduruku ethnic group is among the fifteen most populous in Brazil. However, no publications were found on how CVDs have affected this ethnic group^(11)^.

It is important to note that the modifiable risk factors for SAH imply behavioral issues. Therefore, they need prevention and monitoring strategies. In view of this, the investigation of cardiovascular risk factors among indigenous people has shown to be essential for the establishment of goals and strategies that enable the epidemiological rupture of the risk factor-disease chain in groups living in vulnerability situations^(12)^.

In this sense, the role of nurses in the Multidisciplinary Indigenous Health Teams (*Equipes Multidisciplinares de Saúde Indígena*, EMSI) stands out, as this professional is qualified to early identify risk factors. Having health service management duties and, therefore, being involved in planning, implementation and preventive actions, he assumes a primary role in establishing strategies that provide for the improvement of health care to the indigenous population^(13)^.

This study brings relevant contributions on the behavior of hypertension among indigenous people living in the state of Amazonas, not only because they live in a region with high social vulnerability, present a very low human development index with precarious access to treated water, unavailability of sewage and electricity, but also because they make up the 18% of the Brazilian indigenous population that resides inside or outside demarcated indigenous lands^(11,14)^. The results presented make it possible to strengthen care and health promotion actions, both for the investigated group and for other ethnic groups that live in a similar cultural context.

The objective of this study was to identify the risk factors associated with pre-hypertension and arterial hypertension among Munduruku indigenous people in the Brazilian Amazon.

## Method

### Study design

This is an epidemiological and cross-sectional study, representative of the ethnic group involved, with a quantitative approach. Epidemiology aims to study the health-disease process, by explaining certain facts and events, as well as to analyze the distribution of diseases, harms and health problems. Its purpose is to provide subsidies for making decisions aimed at benefiting the health of the population.

### Data collection place

Data collection was carried out in the villages of Laranjal, Mucajá, Kwatá and Fronteira, located in the Kwatá-Laranjal indigenous land, belonging to the geographical region of the municipality of Borba in the state of Amazonas (AM), Brazil.

### Period

Data collection was carried out from August to September 2018. The average length of stay in each village was nine days.

### Population

The study population consisted of Munduruku indigenous people aged between 18 and 80 years old, of both genders. Historical data refer to being an ethnic group from the Tupi trunk, from the Munduruku linguistic family. Currently, the Munduruku language is undergoing a disuse process. Those who live in the Kwatá-Laranjal indigenous land speak Portuguese^(15)^.

The Kwatá-Laranjal indigenous land belongs to the Special Indigenous Sanitary District (*Distrito Sanitário Especial Indígena*, DSEI) of Manaus (AM) and is made up of 31 villages. It has two base poles, which have as reference the villages of Kwatá (21 villages) and Laranjal (10 villages), accounting for 2,484 Munduruku indigenous people only in Amazonas^(15)^. Access to the Indigenous Land can be through the municipality of Nova Olinda do Norte (AM), through two routes: the first option leaves the port located in the South Zone of Manaus, with approximately 7 hours of travel; the second option is departing from the central port region of Manaus, in a trip lasting approximately 15 hours.

Data from the Socioenvironmental Institute (*Instituto Socioambiental*, ISA), for the entire national territory, show that the Munduruku ethnic group has a population of 14,093 individuals located in the states of Amazonas (East region, Canumã river and close to Transamazônica, Borba municipality), Pará (Southeast region, municipalities of Santarém, Iaituba and Jacareacanga) and Mato Grosso (North region of the municipality of Juara)^(15)^.

### Selection criteria

The study included indigenous people of the Munduruku ethnic group, aged between 18 and 80 years old, living in the villages of Laranjal, Mucajá and Fronteira, located in the state of Amazonas. Those with motor limitations and/or diseases that made it impossible for them to answer any of the research exams were excluded, as well as pregnant women.

### Sample definition

Sample calculation was based on the prevalence of arterial hypertension estimated at 50%^(12)^, margin of error of 5%, confidence interval of 95% and percentage of losses (10%). Based on this calculation, the sample consisted of 459 indigenous of the Munduruku ethnic group, from the Laranjal (n=93), Mucajá (n=129), Kwatá (n=136) and Fronteira (n=101) villages.

For selection, stratified random sampling (unweighted) was used. Each family represented a stratum of the population. In each family (stratum), an individual was drawn to compose the final sample.

### Study variables

Blood pressure was measured with an automatic device (OMRON HBP-1100) validated and calibrated by the Institute of Energy and Nuclear Research (*Instituto de Pesquisas Energéticas e Nucleares*, IPEN), with a cuff of the appropriate size for the arm circumference. The preparation of the indigenous people and the measurement procedures followed the guidelines of the 7^th^ Brazilian Guideline on Arterial Hypertension and the Instruction Manual of the blood pressure monitor, which instructs how to use the equipment correctly^(2)^. For this measurement, the participants were seated comfortably, with their feet flat on the floor and their left arm supported in the direction of the heart. Measurements were taken on this arm and after ten minutes of rest. Before, however, the participants were instructed to empty their bladder and confirm that they had not drunk alcohol or coffee and had not smoked in the last 30 minutes. Three blood pressure measurements were taken and the mean of the last two was used to analyze the data.

Hypertension was defined as systolic blood pressure ≥ 140 and/or diastolic blood pressure ≥ 90 mmHg. Alternatively, hypertension could be self-reported (reported), if the indigenous participant indicated having been diagnosed with hypertension by a physician or if they were taking some antihypertensive medication, regardless of the blood pressure values measured in the interview. Pre-hypertension was defined as systolic blood pressure values of 130-139 mmHg and/ or diastolic blood pressure values of 85-89 mmHg. Blood pressure (pre-SAH and SAH) was classified in stages, following the recommendations of the 7^th^ Brazilian Guideline for Arterial Hypertension^(2)^.

For the anthropometric assessment, a digital bioimpedance scale (OMRON HBF-514C) was used, with a maximum capacity of 150 kilograms (kg); as well a portable stadiometer to check height and inelastic measuring tape to check the neck, waist and hip circumferences. The cutoff points, adopted to indicate increase and/or changes, were as follows: neck circumference ≥ 37 centimeters (cm) for men and ≥ 34 cm for women^(16)^; waist circumference ≥ 102 cm in men and ≥ 88 cm in women and waist/hip ratio (WHR) > 1.00 cm for men and > 0.85 cm for women^(17)^.

The body mass index was classified as follows: low weight (<18.5 kg/m^2^); eutrophic (from 18.5 kilos by height squared - kg/m^2^ to 24.9 kg/m^2^); excess weight (from 25.0 kg/m^2^ to 29.9 kg/m^2^) and obesity (≥ 30.0 kg/m^2^)^(17)^. For the classification of the body fat percentage, the stratification of age group and gender of the indigenous people was considered, as they are different.

Capillary blood glucose was checked using a portable digital device (Accu-Check^®^ glucometer from *Roche Diagnóstica*). For the classification of the blood glucose changes, pre-diabetes was considered with 100 mg/dL125 mg/dL (milligrams *per* deciliter) and diabetes, with ≥ 126 mg/dL^(18)^.

For the measurement of the cholesterol and triglyceride levels, a digital monitor (Accutrend^®^ Plus from *Roche Diagnóstica*) was used. Hypercholesterolemia was considered when ≥ 240 mg/dL and hypertriglyceridemia, with ≥ 200 mg/dL^(19)^.

To check the glycemic and lipid levels, the blood samples were obtained by puncturing the pulp of the participant’s index finger. For this, an individual and disposable puncture device (lancing device) was used.

An interview was also conducted with the indigenous people to collect sociodemographic data and to investigate habits and lifestyles, through semi-structured questions and validated instruments, such as: Alcohol Use Disorder Identification Test (AUDIT)^(20)^ and the International Physical Activity Questionnaire (IPAQ), in its short version^(21)^.

### Instruments used to collect information

A form consisting of closed questions related to the following variables was applied: gender, age, anthropometric data, blood pressure, blood glucose, lipid profile, marital status, income, schooling, occupation, socioeconomic characterization, eating habits, smoking, alcohol consumption, family history of CVDs and level of physical activity.

The socioeconomic classifications were determined according to the Brazilian Economic Classification Criteria^(22)^, which take into account the participants’ housing conditions (number of bathrooms, water source, type of pavement on the street where the house is located and number of domestic servants), number of durable consumer goods, appliances and electronics and schooling levels. Based on this data, the person is classified into one of the following categories: Class A (45-100 points), Class B1 (38-44 points), Class B2 (29-37 points), Class C1 (23-28 points), Class C2 (17-22 points) and Class D-E (0-16 points). The monthly family income was calculated based on the minimum wage, which, at the time of data collection, was R$ 954.00, which corresponded to a value of US$ 234.31.

To estimate the level of physical activity, the IPAQ in its short version was used, with validity and reproducibility in Brazil proven in 2000. The IPAQ is a questionnaire proposed by the World Health Organization (WHO) for the global assessment of physical activity^(21)^. The questions are related to the activities performed in the last seven days before its application (frequency and intensity of the weekly walk, number of days and time to perform moderate activities, number of days and time to perform vigorous activities during the week, and description of sedentary activities).

The level of physical activity of the indigenous people was classified based on the guidance of the IPAQ itself^(21)^: *Very Active*: Vigorous activity: ≥ 5 days/week and ≥ 30 minutes *per* session or Vigorous Activity: ≥ 3 days/week and ≥ 20 minutes *per* session + Moderate Activity or Walking: ≥ 5 days/week and ≥ 30 minutes *per* session. *Active*: Vigorous Activity: ≥ 3 days/week and ≥ 20 minutes *per* session or Moderate Activity or Walking: ≥ 5 days/week and ≥ 30 minutes *per* session or any activity totaling: ≥ 5 days/week and ≥ 150 minutes/ week (walking + moderate activity + vigorous activity). *Irregularly Active*: different types of activities (Walking + Moderate Activity + Vigorous Activity) totaling insufficient frequency and duration for the individual to be considered active. *Sedentary*: People who did not perform any physical activity for at least 10 continuous minutes during the week.

The Alcohol Use Disorder Identification Test (AUDIT) instrument was used to calculate the consumption of alcoholic beverages, validated for the Portuguese language^(23)^, whose objective is to identify disorders due to alcohol consumption. The ten questions of this instrument explore the use, dependence and problems related to alcohol. The AUDIT’s first question is about the consumption frequency and is answered on a scale ranging from 0 (never) to 4 (four or more times a week). The score ranges from 0 to 40 but, in a score up to 7, consumption is low risk; from 8 to 15 points, consumption is risky and use is harmful; finally, highrisk consumption, equal to or greater than 16, indicates probable dependence. The ten items cover three theoretical domains: frequency of alcohol consumption, dependence on alcohol consumption, and negative consequences of alcohol consumption. AUDIT does not make any diagnosis, but indicates the probable cases of dependence^(23)^.

### Data collection

Data collection was conducted by nurses trained to perform each stage.

The data were collected in the morning, in view of the need for fasting. The indigenous people were previously instructed to have their last meal until 10 pm the day before collection and not to eat when they woke up. In all the villages, collection took place in the same way: first the Indigenous Health Agents (*Agentes Indígenas de Saúde*, AIS) provided the registration of the families in each village and, based on this list, the sample was drawn. Immediately after that, it was assessed whether all the people selected met the inclusion criteria; if this was not the case, a new draw was carried out to replace the excluded person. After this procedure, the invitation was made individually. The indigenous person who agreed to participate in the study was previously informed about the time of collection and the conditions necessary to carry out the examinations and anthropometric measurements.

The indigenous people who presented changes in blood pressure and/or in any of the tests performed were referred for appointments at the Health Base.

### Data treatment and analysis

The analyses of the data collected were carried out in the R software, version 3.5.1. The continuous variables were presented based on their means and standard deviation; the categorical variables, by means of absolute and relative frequencies. The variables were compared using hypothesis tests. With the continuous variables, the Kruskall-Wallis test was used; for categorical ones, Fisher’s Exact test. The significance level was set at 5% and p-value≤0.05. The Wald test was used in multinomial regression.

To verify the association between the dependent variables (pre-hypertension and hypertension) and the independent variables of the study, the likelihood ratios were estimated by Odds Ratio (OR) based on the multinomial regression model and respective 95% confidence intervals (CIs). For being a multifactorial phenomenon, the variables were grouped in blocks (demographic, economic, health status and behavioral) and analyzed hierarchically^(24)^.

### Ethical aspects

The study was approved by the National Research Ethics Commission (CAAE No. 74361617.2.0000.5020) in accordance with Opinion No. 251,369. For entry into indigenous land, authorization was requested from the National Indian Foundation (No. 43/AAEP/PRES/2018), from the Ministry of Justice and, also, from the leaders of the Kwatá-Laranjal Indigenous Land. All the indigenous people who agreed to participate in the study signed the Free and Informed Consent Form.

## Results

Among the 459 indigenous people investigated, the prevalence of altered blood pressure levels, obtained through casual measurement, was 10.2% for values suggestive of hypertension and 4.1% for prehypertension.

Table 1 shows the univariate analysis of the participants’ sociodemographic, anthropometric, metabolic and lifestyle variables according to the pressure levels that indicate normal pressure, SAH and pre-SAH. It is noteworthy that most of the participants were male (57.1%), that their mean age was 36.6 (±14.7) years old and that nearly 9.6% were illiterate. As for family income, almost all (83.7%) were in a situation of social vulnerability (belonging to social classes D and E). A high percentage of participants (61.7%) reported having a social benefit offered by the government as their only source of income.

**Table 1:** Profile of the blood pressure levels of the Munduruku indigenous people. Municipality of Borba, AM, Brazil, 2018

Variables	Normal	Pre-hypertension	Hypertension	Total	p- value^§^
	n (%) 393 (85.6)	n (%) 19 (4.1)	n (%) 47 (10.2)	n (%) 459 (100)	
*Sociodemographic Factors*					
Gender					
Female	167 (42.5)	6 (31.6)	24 (51.1)	197 (42.9)	0.317
Male	226 (57.5)	13 (68.4)	23 (48.9)	262 (57.1)	
Age (In years old, Mean, SD)*	34.2 ± 12.9	36.4 ± 12.0	56.7 ± 15.1	36.6 ± 14.7	**< 0.001**
Marital Status					
Has a partner	269 (68.4)	13 (68.4)	30 (63.8)	312 (68.0)	0.813
No partner	124 (31.6)	6 (31.6)	17 (36.2)	147 (32.0)	
Schooling					
Illiterate	22 (5.6)	2 (10.5)	20 (42.6)	44 (9.6)	**< 0.001**
Elementary School 1-2	149 (37.9)	8 (42.1)	18 (38.3)	175 (38.1)	
High School	166 (42.2)	6 (31.6)	5 (10.6)	177 (38.6)	
Higher Education or Graduate	56 (14.2)	3 (15.8)	4 (8.5)	63 (13.7)	
Family Income (minimum wage)^†^					
< 1	175 (46.4)	9 (50.0)	11 (24.4)	195 (44.3)	**< 0.001**
Up to 1	19 (5.0)	2 (11.1)	11 (24.4)	32 (7.3)	
Up to 2	113 (30.0)	4 (22.2)	12 (26.7)	129 (29.3)	
Up to 3	40 (10.6)	2 (11.1)	7 (15.6)	49 (11.1)	
>4	30 (8.0)	1 (5.6)	4 (8.9)	35 (8.0)	
Social benefit					
Yes	255 (64.9)	12 (63.2)	16 (34.0)	283 (61.7)	**< 0.001**
No	138 (35.1)	7 (36.8)	31 (66.0)	176 (38.3)	
Economic Classification^‡^					
B2 (29-37 points)	5 (1.3)	1 (5.3)	1 (2.1)	7 (1.5)	0.164
C1-C2 (17-28 points)	64 (16.3)	1 (5.3)	3 (6.4)	68 (14.8)	
D-E (0-16 points)	324 (82.4)	17 (89.5)	43 (91.5)	384 (83.7)	
*Anthropometric Factors*					
Neck Circumference					
Normal	149 (37.9)	3 (15.8)	9 (19.1)	161 (35.1)	**0.008**
Increased	244 (62.1)	16 (84.2)	38 (80.9)	298 (64.9)	
Waist Circumference					
Normal	261 (66.4)	9 (47.4)	11 (23.4)	281 (61.2)	**< 0.001**
Increased	65 (16.5)	4 (21.1)	14 (29.8)	83 (18.1)	
Substantially increased	67 (17.0)	6 (31.6)	22 (46.8)	95 (20.7)	
Waist-Hip Ratio					
Normal	226 (57.5)	9 (47.4)	7 (14.9)	242 (52.7)	**< 0.001**
Increased	167 (42.5)	10 (52.6)	40 (85.1)	217 (47.3)	
Body Mass Index					
Eutrophic (up to 24.9 kg/m2)	201 (51.1)	5 (26.3)	11 (23.4)	217 (47.3)	**< 0.001**
Excess weight (25 to 29.9 kg/m2)	152 (38.7)	7 (36.8)	17 (36.2)	176 (38.3)	
Obesity (>=30 kg/m2)	40 (10.2)	7 (36.8)	19 (40.4)	66 (14.4)	
Body Fat					
Low or Normal	164 (41.7)	3 (15.8)	9 (19.1)	176 (38.3)	**< 0.001**
High	112 (28.5)	7 (36.8)	11 (23.4)	130 (28.3)	
Very High	117 (29.8)	9 (47.4)	27 (57.4)	153 (33.3)	
*Metabolic Factors*					
Capillary Glycaemia					
Normal	59 (15.0)	1 (5.3)	2 (4.3)	62 (13.5)	**< 0.001**
Pre-diabetes	297 (75.6)	12 (63.2)	32 (68.1)	341 (74.3)	
Diabetes	37 (9.4)	6 (31.6)	13 (27.7)	56 (12.2)	
Triglycerides					
Desirable	250 (63.6)	10 (52.6)	23 (48.9)	283 (61.7)	0.01
Borderline	71 (18.1)	2 (10.5)	6 (12.8)	79 (17.2)	
High	72 (18.3)	7 (36.8)	18 (38.3)	97 (21.1)	
Total Cholesterol					
Desirable	319 (81.2)	16 (84.2)	27 (57.4)	362 (78.9)	**< 0.001**
Borderline	58 (14.8)	2 (10.5)	12 (25.5)	72 (15.7)	
High	16 (4.1)	1 (5.3)	8 (17.0)	25 (5.4)	
*Habits and lifestyle*					
Smoke/Smoked					
No	99 (25.2)	2 (10.5)	3 (6.4)	104 (22.7)	**0.006**
Yes	294 (74.8)	17 (89.5)	44 (93.6)	355 (77.3)	
Physical Activity					
Sedentary	21 (5.3)	1 (5.3)	13 (27.7)	35 (7.6)	**< 0.001**
Irregularly Active	100 (25.4)	4 (21.1)	19 (40.4)	123 (26.8)	
Active/Very Active	272 (69.2)	14 (73.7)	15 (31.9)	301 (65.6)	
Alcohol Consumption					
Low-risk consumption	42 (27.8)	3 (50.0)	1 (33.3)	46 (28.8)	0.492
Risk use (harmful or probable dependence)	109 (72.2)	3 (50.0)	2 (66.7)	114 (71.2)	

* SD = Standard Deviation;

† Current minimum wage = R$ 954.00, Brazil, 2018, equivalent to US$ 234.31;

‡ Brazil Economic Classification Criterion;

§ p-value = Qualitative variables: Fisher's Exact test and Kruskall-Wallis test for continuous variables

Among the Munduruku indigenous people, 64.9% presented an increase in neck circumference; 38.8%, increased waist circumference; and more than 52% were overweight. These data corroborate the high percentage of indigenous people classified as having a high body fat index: high (28.3%) and very high (33.3%). Regarding the levels of triglycerides and cholesterol, the altered values were around 21.1% and 5.4%, respectively. It is noteworthy that 86.5% of the participants presented high levels of capillary blood glucose and that more than 70% stated that they were smokers and consumed alcohol at levels considered to be harmful risk or probable dependence. In the case of physical activity, 65.6% were classified as active or very active (Table 1).

Table 2 shows the multivariate analysis using a multinomial logistic regression model of all the participants’ sociodemographic, anthropometric, metabolic and lifestyle variables, according to the altered blood pressure levels suggestive of SAH and pre-SAH.

**Table 2:** Anthropometric, metabolic and lifestyle indicators of the Munduruku indigenous people (n=459), according to high blood pressure levels. Municipality of Borba, AM, Brazil, 2018

Variables	Pre-hypertension vs Normal Gross OR^†^ [95% CI]	p-value^‡^	Hypertension vs Normal Gross OR^†^ [95% CI]	p-value^‡^
*Sociodemographic Factors*				
Gender				
Female	1.00	-	1.00	-
Male	1.60[0.60;4.30]	0.350	0.71[0.39;1.30]	0.264
Age (Years old, Mean [SD])	1.01[0.98;1.05]	0.464	1.10[1.08;1.13]	**<0.001**
Marital Status				
Has a partner	1.00	-	1.00	-
No partner	1.00[0.37;2.70]	0.998	1.23[0.65;2.31]	0.522
Schooling				
Illiterate	1.00	-	1.00	-
Elementary School 1-2	0.59[0.12;2.97]	0.523	0.13[0.06;0.29]	**<0.001**
High School	0.40[0.08;2.09]	0.277	0.03[0.01;0.10]	**<0.001**
Higher or Postgraduate	0.59[0.09;3.77]	0.577	0.08[0.02;0.26]	**<0.001**
Family income (minimum wage)				
< 1 wage	1.00	-	1.00	-
Up to 1	2.05[0.41;10.17]	0.381	9.21[3.52;24.07]	**<0.001**
Up to 2	0.69[0.21;2.29]	0.542	1.69[0.72;3.96]	0.227
Up to 3	0.97[0.20;4.67]	0.972	2.78[1.02;7.63]	**0.046**
> 4 minimum wages	0.65[0.08;5.30]	0.686	2.12[0.63;7.10]	0.222
Receives social benefit				
Yes	1	-	1	-
No	1.08[0.41;2.80]	0.878	3.58[1.89;6.78]	**<0.001**
Economic classification				
B2	1	-	1	-
C1-C2	0.08[0.00;1.45]	0.087	0.23[0.02;2.69]	0.244
D-E	0.26[0.03;2.38]	0.234	0.66[0.08;5.83]	0.712
*Anthropometric Factors*				
Neck circumference				
Normal	1	-	1	-
Increased	3.26[0.93;11.37]	0.064	2.58[1.21;5.48]	**0.014**
Waist circumference				
Normal	1	-	1	-
Increased	1.79[0.53;5.98]	0.347	5.11[2.22;11.78]	**<0.001**
Substantially increased	2.60[0.89;7.55]	0.080	7.79[3.60;16.86]	**<0.001**
Waist/hip ratio				
Normal	1	-	1	-
Increased	1.50[0.60;3.78]	0.386	7.73[3.38;17.69]	**<0.001**
BMI*				
Eutrophic (Up to 24.9 kg/m2)	1	-	1	-
Excess weight (25 to 29.9 kg/m2)	1.85[0.58;5.95]	0.301	2.04[0.93;4.49]	0.075
Obesity (>=30 kg/m2)	7.03[2.13;23.28]	0.001	8.68[3.84;19.64]	**<0.001**
Body Fat				
Low or Normal	1	-	1	-
High	3.42[0.87;13.50]	0.080	1.79[0.72;4.46]	0.212
Very High	4.21[1.11;15.87]	0.034	4.21[1.91;9.27]	<0.001
*Metabolic Factors*				
Capillary Glycaemia				
Normal	1	-	1	-
Pre-diabetes	2.38[0.30;18.70]	0.408	3.18[0.74;13.63]	0.119
Diabetes	9.57[1.11;82.73]	0.040	10.37[2.21;48.56]	**0.003**
Triglycerides				
Desirable	1	-	1	-
Borderline	0.70[0.15;3.29]	0.655	0.92[0.36;2.34]	0.859
High	2.43[0.89;6.61]	0.082	2.72[1.39;5.31]	**0.003**
Total Cholesterol				
Desirable	1	-	1	-
Borderline	0.69[0.15;3.08]	0.625	2.44[1.17;5.10]	**0.017**
High	1.25[0.15;10.01]	0.836	5.91[2.32;15.05]	**<0.001**
*Habits and lifestyle*				
Smokes				
No	1	-	1	-
Yes	2.87[0.65;12.69]	0.164	4.94[1.50;16.28]	**0.009**
Physical activity				
Sedentary	1	-	1	-
Irregularly Active	0.84[0.09;7.90]	0.879	0.31[0.13;0.72]	**0.006**
Active/Very Active	1.08[0.14;8.62]	0.941	0.09[0.04;0.21]	**<0.001**
Consumption of Alcohol Beverages				
Low-risk consumption	1	-	1	-
Risk use (harmful or probable dependence)	0.39[0.07;1.99]	0.254	0.77[0.07;8.73]	0.834

* BMI = Body Mass Index;

† OR = Odds Ratio;

‡ p-value = Wald test. Note: Conventional sign used: Numeric data equal to zero not resulting from rounding

In relation to the sociodemographic and economic variables of the participants classified as pre-hypertensive and hypertensive, a positive association was observed with regard to age, revealing that for every year in increasing age, the chance of developing SAH increased by 10% (OR=1.0; 95% CI=1.08-1.13). When compared to those with less schooling, the participants who completed high school presented up to 97% fewer chances of developing SAH (OR=0.03; 95% CI=0.01-0.10). Those with incomes up to a minimum wage had 9 times more chances of developing SAH (OR=9.21; 95% CI=3.5224.07). As for the social benefit, those who indicated receiving some modality were 3 times more likely to develop SAH (OR=3.58; 95% CI=1.89-6.78).

It is noteworthy that the increased circumferences of the neck (OR=2.58; 95% CI=1.21-5.48), the waist (OR=7.79; 95% CI=3.60-16.86) and WHR (OR=7.73; 95% CI=3.38-17.69) substantially increased (from 2 to 7 times) the chance of developing hypertension, as well as those classified as obese, whose chance increased 7 times for pre-SAH (OR=7.03; 95% CI=2.13-23.28) and 8 times for SAH (OR=8.68; 95% CI=3.84-19.64). Another important anthropometric indicator reveals that indigenous people classified as having very high body fat were 4 times more likely to develop pre-hypertension and hypertension (OR=4.21; 95% CI=1.91-9.27).

In relation to capillary blood glucose, those classified as diabetic (12.2%) were 9 times more likely to have pre-SAH (OR=9.57; 95% CI=1.1182.73) and 10 times for SAH (OR=10.37; 95% CI=2.21-48.56). The indigenous individuals with triglycerides (OR=2.72; 95% CI=1.39-5.31) and high cholesterol (OR=5.91; 95% CI=2.3215.05) presented 2 and 5 times more chances of presenting SAH, respectively. Those who were classified as with borderline cholesterol (OR=2.44; 95% CI=1.17-5.10) were also 2 times more likely to develop SAH.

As for the smoking habit, it was positively associated with SAH, presenting 4 times more chance for the risk of developing it (OR=4.94; 95% CI=1.50-16.28). On the other hand, practicing physical activity proved to be a protective factor against SAH, protecting up to 69% (OR=0.31; 95% CI=0.13-0.72) of those who were irregularly active and up to 99% of those who were active (OR=0.09; 95% CI=0.04-0.21), when compared to the sedentary individuals.

As for the marital status, economic classification and consumption of alcoholic beverages variables, it is worth mentioning that they did not present a positive association with pre-SAH and SAH.

For the construction of the multiple regression model, the effect size and the p-value of each variable were analyzed in each of the blocks of variables (demographic, economic, health and behavioral). The significant variables of each block were separated in hierarchical blocks for the analysis. In some cases, as in the health status block, the variables considered were collinear (highly associated with each other). In this case, it was necessary to choose the most important variables (those with greater effect), to proceed with the multiple analysis.

Therefore, the following variables remained in the model: gender, age, increased waist circumference and substantially increased waist circumference variables (Table 3). The result allowed inferring that men were 4 times more likely to develop pre-SAH (OR=1.65; 95% CI=0.65-4.21) and that, for each one-year-old increase, there was a 9% increase in the chance of hypertension (OR=1.09; 95% CI=1.06-1.12). In relation to waist, those with increased circumference were 3 times more likely to develop SAH (OR=3.89; 95% CI=1.43-10.54), while those with substantially increased circumference were 7 times more likely to present pre-SAH (OR=7.82; 95% CI=1.80-34.04) and 5 times more likely to have SAH (OR=5.46; 95% CI=1.78-16.75).

**Table 3:** Final risk model for the development of SAH in the Munduruku indigenous people (n=459). Municipality of Borba, AM, Brazil, 2018

Variables	Pre-hypertension vs Normal Adjusted OR [95% CI]	p-value*	Hypertension vs Normal Adjusted OR [95% CI]	p-value*
Intercept	1	-	1	-
Gender (male)	4.67[1.28 – 17.02]	0.019	1.65[0.65 – 4.21]	0.292
Age (years old)	1.00[0.96 – 1.03]	0.811	1.09[1.06 – 1.12]	**<0.001**
Waist (Increased)	314[0.84 – 11.68]	0.088	3.89[1.43 – 10.54]	**0.008**
Waist (Substantially increased)	7.82[1.80 – 34.04]	0.006	5.46[1.78 – 16.75]	**0.003**

* p-value = Qualitative variables, Fisher's Exact test; and Kruskall-Wallis for the continuous variables. Note: Conventional sign used: Numeric data equal to zero not resulting from rounding

## Discussion

In this study, it was possible to present data more representative of the men, who were the majority. This result confirms data from the demographic census, which indicates that the largest proportion of the male population lives in rural areas^(25)^. On the other hand, it differs from other studies, which generally present a higher proportion of women^(8,26)^. This representativeness, however, was coincidental, as sample selection was at random.

In this study, men were more vulnerable to developing hypertension. A similar finding was found among Krenak indigenous people, whose estimated prevalence in males was higher when compared to females (31.2% x 27.6%). The authors draw the call attention to other factors, such as age, search for medical diagnosis and adherence to treatment, among others, that can differentiate the frequency of the disease between the genders^(27)^. A study of indigenous populations in Chile also showed that men were more likely to have high systolic and diastolic blood pressure values when compared to women^(26)^.

Although the participants in this study were predominantly young adults, age proved to be a strong cardiovascular risk factor. Studies carried out with different ethnic groups have shown a positive association between aging and the prevalence of SAH^(2,9)^. This finding is similar to that identified in a study with a tribal population in India, revealing that the prevalence of hypertension increased with age^(28)^.

The social vulnerability and low schooling found among the Munduruku under study were similar to those of other ethnic groups, which also showed the direct impact of the social and economic determinants on the mechanisms and distribution of diseases, including cardiovascular ones^(5,29)^.

The prevalence of SAH (10.2%) found among the Munduruku investigated in a systematic review study^(9)^ was lower than that of non-indigenous people and slightly higher when compared to other ethnic groups, such as Terena, Suyá, Guaraní Mbyá, Kuikuro, Parkatêjê and indigenous individuals from Parque do Xingu, Suruí and Khisêdjê, who presented a combined prevalence of 6.2%. In the analysis by period (1970 to 2014), the study showed that the chance of an indigenous person having the disease increased by up to 12%.

In turn, other research studies have identified prevalence values for SAH close to and even above the national mean in two different ethnic groups: the Kaingangs (in Santa Catarina - SC) and the Mura (AM); the prevalence values for SAH in these groups were 53.2% and 26.6%, respectively^(10,12)^.

The pressure values indicative of pre-SAH also deserve attention, as they are borderline and can put the person at risk of contracting not only SAH but other cardiac comorbidities. A study carried out with a group of indigenous people in Canada revealed prevalence of pre-hypertension (17.7%) and hypertension (21.7%), in addition to a warning of undiagnosed hypertension. This result indicates the urgent need for screening to prevent the serious complications and adverse health problems associated with high blood pressure, which can reduce quality of life^(30)^.

The risk for the incidence of cardiovascular diseases, ischemic heart disease and stroke is higher in prehypertensive individuals [Systolic Blood Pressure (SBP) ≥130-139 or Diastolic Blood Pressure (DBP) ≥ 85-89 mmHg], when compared to those who present blood pressure values considered normal^(2)^. In the case of the indigenous people in this study, the evidence points to the need to establish strict blood pressure monitoring for pre-hypertensive individuals, considering the greater chance of developing SAH in subsequent years.

In this scenario, the role of Nursing stands out, as the nurse has specific professional skills and competence to act in the planning, implementation and evaluation of strategies aimed at education in health^(31)^.

It is noteworthy that, in the context of indigenous health, the nurse’s focus is to promote effective actions, to allow for greater mobilization of the ethnic groups and to recover good self-care practices, enabling the resumption of healthy habits without necessarily having to interrupt dialog with the non-indigenous society^(32)^. Self-care, in turn, requires determination on the part of the individual who already has some cardiovascular risk factor. Therefore, the nurse needs to support and encourage the patients’ self-determination, seeking to identify the preferences that the individuals themselves have for self-care^(33)^.

Regarding the anthropometric characteristics, as there is no specific standardization for the indigenous populations, national cutoff points were adopted as a parameter of normality and alterations. In the multivariate analysis, the prevalence values of obesity, as well as those of increased waist and neck circumferences and of the WHR were positively associated with the chance of developing SAH. This finding is worrying both for the group under study and for other ethnicities. In addition to this, it reinforces the need for studies that show the risk factors for CNCDs, such as obesity, which are disproportionately more prevalent among some ethnic minorities, people of lower socioeconomic status and rural populations in most Latin American countries^(7,34)^.

Although most of the indigenous villagers have a routine focused on agricultural activities, the proximity to cities, in rural and urban areas, seems to have a negative influence on the eating habits and lifestyle. This reality has strongly contributed to the increase in the prevalence of excess weight and obesity, considered important in the development of chronic diseases, such as SAH, insulin resistance, DM and dyslipidemia^(6,35)^. The variety and ease of access to industrialized products by indigenous people in Brazil and other parts of the world have been shown to be directly related to the increase in body weight^(6)^.

A number of studies relate the high prevalence of dyslipidemia among indigenous people to the important contact with urbanization^(36-37)^. The lipid profile of the participants in this research was high, similar to that found among the Xavante ethnic group^(36)^. As for capillary glycaemia, although it is not positively associated with the risk of pre-SAH and/or SAH in the final model of the analysis of this study, it is still considered as an alarming data, since SAH and DM, together, are the main causes of morbidity and mortality^(37)^.

In relation to smoking, it is important to highlight that it is the main cause of preventable deaths. Even considering that tobacco use is an old practice among indigenous people, it is necessary to intensify actions to combat smoking in these populations, as this habit is an important risk factor for cardiovascular diseases^(38)^.

More than half of the Munduruku reported smoking. It is interesting to note that, among hypertensive patients, the majority (93.6%) were also smokers. High prevalence of smoking was found in different ethnic groups in Brazil and Chile^(10,26)^.

With regard to physical activity, it was shown to be protective for the participants classified as irregularly active, active or very active. Hypertensive patients were the most sedentary. The presence of physical inactivity in other ethnic groups has been associated with the presence of SAH, increased age and obesity^(8)^.

Although the consumption of alcoholic beverages did not indicate any risk for SAH in the group under study, the percentage of indigenous people in the zone of harmful risk use or probable dependence is noteworthy. This data raises concerns, since chronic and high consumption of alcoholic beverages (more than 31 grams *per* day - g/ day) increases blood pressure consistently^(2)^.

Studies that evaluated the permanence and changes in the eating habits of agricultural families indicated that the general trends of changes in these habits had repercussions on the habits of those who lived in rural areas, intensifying them due to the proximity of urban areas, due to the greater possibility of incorporating industrialized products^(39-40)^. The results herein found pointed to the need for education in health on the risk factors for cardiovascular diseases, specifically for the prevention of SAH and pre-SAH. They also included guidelines and promotion of activities aimed at protective health practices, such as physical exercise, adequate food and even care with medication therapy for the hypertensive person.

In addition to this, special attention from the EMSI is suggested regarding blood pressure values suggestive of pre-hypertension, as the prevalence found in this study indicates the need for an effective intervention to prevent the manifestation of the disease. It is necessary to emphasize that the lack of specific data for this variable made it impossible to make a more in-depth comparison, being considered a study limitation. It is suggested that future research studies include in their analyses pressure values suggestive of pre-hypertension as a risk factor not only for SAH, but also for other cardiovascular comorbidities.

In this scenario, coping strategies need to be linked to a public policy based on full respect for cultural diversity. At the same time, they must enable greater integration and agreement among the protagonists of this process of change, which involve active participation of the community in harmony with the multidisciplinary health team, highlighting the role of the nurse, as their skills and abilities allow mediating practices of care and selfcare aimed at promoting better quality of life and health.

## Conclusion

In this study, among the Munduruku indigenous people of the Brazilian Amazon, it was identified that men were more vulnerable to developing hypertension, age was shown to be a strong cardiovascular risk factor and increased and substantially increased waist circumference increased the chance of an indigenous person presenting pre-hypertension and arterial hypertension, respectively. The profile found is considered to be the result of sociocultural, economic and environmental changes among the Munduruku.

## References

[1] World Health Organization (2018). Noncommunicable diseases country profiles 2018 [Internet].

[2] Barroso WKS, Rodrigues CIS, Bortolotto LA, Gomes MAM, Brandão AA, Feitosa ADM (2020). Diretrizes Brasileiras de Hipertensão Arterial –
2020.. Arq Bras Cardiol..

[3] Mulerova T, Uchasova E, Ogarkov M, Barbarash O. (2020). Genetic forms and pathophysiology of essential arterial
hypertension in minor indigenous peoples of Russia.. BMC Cardiovasc Disord..

[4] Mills KT, Stefanescu A, He J. (2020). The global epidemiology of hypertension.. Nat Rev Nephrol..

[5] Ministério da Saúde (BR) (2020). Secretaria de Vigilância em Saúde, Vigitel Brasil 2019. Vigilância de
fatores de risco e proteção para doenças crônicas por inquérito telefônico:
estimativas sobre frequência e distribuição sociodemográfica de fatores de
risco e proteção para doenças crônicas nas capitais dos 26 Estados
brasileiros e no Distrito Federal em 2019 [Internet].

[6] Armstrong AC, Ladeia AMT, Marques J, Armstrong DMFO, Silva AML, Morais JC (2018). Urbanization is Associated with Increased Trends in
Cardiovascular Mortality Among Indigenous Populations: the PAI
Study.. Arq Bras Cardiol..

[7] Collaboration NRF. (2019). Rising rural body-mass index is the main driver of the global
obesity epidemic in adults.. Nature..

[8] Mendoza-Caamal EC, Barajas-Olmos F, García-Ortiz H, Cicerón-Arellano I, Martínez-Hernández A, Córdova EJ (2020). Metabolic syndrome in indigenous communities in Mexico: a
descriptive and cross-sectional study.. BMC Public Health..

[9] Souza ZA, Ferreira AA, Santos B, Pierin AMG. (2015). Hypertension prevalence among indigenous populations in Brazil: a
systematic review with meta-analysis.. Rev Esc Enf USP.

[10] Souza ZA, Ferreira AA, Santos J, Meira KC, Pierin AMG. (2018). Cardiovascular risk factors with an emphasis on hypertension in
the Mura Indians from Amazonia.. BMC Public Health..

[11] (2010). Instituto Brasileiro de Geografia e Estatística. Os indígenas no censo
demográfico [Internet].

[12] Araújo GA, Mendonça JN, Julião KM, Almeida LL, Aquino RL, Paula RM (2020). Hipertensão arterial sistêmica: um panorama de grupos vulneráveis
de diferentes regiões do Brasil no período de 2005 a 2018.. Braz J Develop..

[13] Viana JA, Cipriano DM, Oliveira MC, Carneiro AMCT, Sá Ribeiro R, Oliveira Feitosa M (2020). A atuação do enfermeiro na saúde indigena: uma análise
integrativa da literatura/Nurses’ performance in indigenous health: an
integrative analysis of the literature.. Braz J Health Rev..

[14] Codeço CT, Villela D, Coelho F, Bastos LS, Gomes MF, Cruz OG (2020). Estimativa de risco de espalhamento da COVID-19 no Brasil e o impacto no
sistema de saúde e população por microrregião [Internet].

[15] Scopel D, Dias R, Langdon EJ. (2018). A cosmografia Munduruku em movimento: saúde, território e
estratégias de sobrevivência na Amazônia brasileira.. Bol Mus Para Emílio Goeldi Ciênc Hum..

[16] Kini S, Shivalli S, Kulkarni V, Mithra P, Kumar N. (2020). Neck Circumference as an Indicator of Obesity and its Comparison
with Body Mass Index and Waist Circumference in Coastal
Karnataka.. Indian J Public Health..

[17] Silveira EA, Pagotto V, Barbosa LS, Oliveira C, Pena GG, Velasquez-Melendez G. (2020). Accuracy of BMI and waist circumference cut-off points to predict
obesity in older adults.. Ciênc Saúde Coletiva..

[18] Forti AC, Pires AC, Pittito BA, Gerchman F, Oliveira JEP, Zajdenverg L (2020). Diretrizes da Sociedade Brasileira de Diabetes 2019-2020
[Internet].

[19] Faludi AA, Izar MCO, Saraiva JFK, Chacra APM, Bianco HT, Afiune A (2017). Atualização da diretriz brasileira de dislipidemias e prevenção
da aterosclerose - 2017.. Arq Bras Cardiol..

[20] Fagundes LC, Paz CJR, Freitas DA, Barbosa HA, Soares WD. (2020). Consumo de álcool entre universitários na cidade de Montes
Claros-MG.. Arq Catarin Med. [Internet].

[21] Matsudo S, Araujo T, Matsudo V, Andrade D, Andrade E, Oliveira LC (2001). Questionário internacional de atividade física (IPAQ): estudo de
validade e reprodutibilidade no Brasil.. Rev Bras Ativ Fís Saúde..

[22] Kamakura W, Mazzon JA. (2016). Critérios de estratificação e comparação de classificadores
socioeconômicos no Brasil.. Rev Adm Emp..

[23] Araújo Sousa KP, Medeiros ED, Medeiros PCB. (2020). Validity and reliability of the Alcohol Use Disorders
Identification Test (AUDIT) in students of a Brazilian
university.. Cienc Psicol..

[24] Victora CG, Huttly SR, Fuchs SC, Olinto M. (1997). The role of conceptual frameworks in epidemiological analysis: a
hierarchical approach.. Int J Epidemiol..

[25] Bastos JL, Santos RV, Cruz OG, Longo LAFB, Silva LO. (2017). Características sociodemográficas de indígenas nos censos
brasileiros de 2000 e 2010: uma abordagem comparativa.. Cad Saúde Pública..

[26] Fernández CI. (2020). Nutrition Transition and Health Outcomes Among Indigenous
Populations of Chile.. Curr Dev Nutr..

[27] Chagas CA, Castro T, Leite MS, Viana MACBM, Beinner MA, Pimenta AM. (2019). Estimated prevalence of hypertension and associated factors in
Krenak indigenous adults in the state of Minas Gerais,
Brazil.. Cad Saúde Pública..

[28] Anand N, Hussain S. (2020). Prevalence of Hypertension & Associated Risk Factors among
Tribal Population in a Rural Community of Katihar.. J Evol Med Dental Sci..

[29] Toledo NN, Almeida GS, Matos MMM, Balieiro AAS, Martin LC, Franco RJS (2020). Cardiovascular risk factors: differences between ethnic
groups.. Rev Bras Enferm..

[30] Spurr S, Bally J, Bullin C, Allan D, McNair E. (2020). The prevalence of undiagnosed Prediabetes/type 2 diabetes,
prehypertension/hypertension and obesity among ethnic groups of adolescents
in Western Canada.. BMC Pediatrics..

[31] Costa DAS, Silva RF, Lima VV, Ribeiro ECO. (2018). Diretrizes curriculares nacionais das profissões da Saúde
2001-2004: análise à luz das teorias de desenvolvimento
curricular.. Interface (Botucatu)..

[32] Martins JCL, Martins CL, Oliveira LSS. (2020). Attitudes, knowledge and skills of nurses in the Xingu Indigenous
Park.. Rev Bras Enferm..

[33] van Wissen K, Blanchard D. The ‘work’ of self-care for people with cardiovascular disease
and prediabetes: An interpretive description. Int J Nurs Stud..

[34] Coimbra CE, Tavares FG, Ferreira AA, Welch JR, Horta BL, Cardoso AM Socioeconomic determinants of excess weight and obesity among
Indigenous women: findings from the First National Survey of Indigenous
People’s Health and Nutrition in Brazil.. Public Health Nutr..

[35] Zorena K, Jachimowicz-Duda O, Ślęzak D, Robakowska M, Mrugacz M. (2020). Adipokines and obesity. Potential link to metabolic disorders and
chronic complications. Int J Mol Sciences..

[36] Soares LP, Fabbro ALD, Silva AS, Sartorelli DS, Franco LF, Kuhn PC (2018). Cardiovascular risk in Xavante indigenous
population.. Arq Bras Cardiol..

[37] Francisco PMSB, Segri NJ, Borim FSA, Malta DC. Prevalência simultânea de hipertensão e diabetes em idosos brasileiros:
desigualdades individuais e contextuais.

[38] Jacondino CB, Schwanke CHA, Closs VE, Gomes I, Borges CA, Gottlieb MGV. (2019). Association of smoking with redox biomarkers and cardiometabolic
risk factors in elder individuals.. Cad Saúde Coletiva..

[39] Maluf RS, Zimmermann SA. (2020). Antigos e novos hábitos na alimentação de famílias agrícolas de
Chapecó e região.. Estudos Soc Agricultura..

[40] Barbosa IEB, Fonseca AR, Souza FC, Andrade ENM, Silva CC, Pinheiro BR (2021). Saúde do adulto indígena com ênfase no sobrepeso e a obesidade em
excesso.. Rev Eletr Acervo Enferm..

